# Evaluating Causal Claims in Plant Developmental Metabolism: A When–Where–How Framework

**DOI:** 10.3390/plants15172604

**Published:** 2026-08-26

**Authors:** Xiangfei Cheng, Yanan Zhao, Leidi Liu, Yaning Li, Ruohang Zhao, Zhiyao Yang, Xinci Hao, Zhongling Yang, Chengde Yu, Chengming Fan, Zhifang Li

**Affiliations:** 1 Center for Molecular Interactions and Translational Applications, School of Life Sciences, Henan University, Kaifeng 475004, China; chengxf@henu.edu.cn (X.C.); ynzhao@henu.edu.cn (Y.Z.); liuleidi@henu.edu.cn (L.L.); liyaning1224@163.com (Y.L.); 18836469082@163.com (R.Z.); yangzhiyao@henu.edu.cn (Z.Y.); 15560200725@163.com (X.H.); yuchengde@henu.edu.cn (C.Y.); 2 State Key Laboratory of Cotton Bio-Breeding and Integrated Utilization, School of Life Sciences, Henan University, Kaifeng 475004, China; 3 International Joint Research Laboratory for Global Change Ecology, Laboratory of Biodiversity Conservation and Ecological Restoration, School of Life Sciences, Henan University, Kaifeng 475004, China; yang_zhl06@126.com; 4 State Key Laboratory of Seed Innovation, Institute of Genetics and Developmental Biology, Chinese Academy of Sciences, Beijing 100101, China

**Keywords:** developmental competence, causal inference, metabolic state, source–sink allocation, spatial metabolism, trehalose 6-phosphate, SnRK1–TOR signaling, phloem transport, specialized metabolism, AI-assisted breeding

## Abstract

Metabolic changes are often described as drivers of plant development even when the evidence establishes only association or a permissive requirement. This review separates biological role from causal status and evaluates claims across seven dimensions: association, necessity, localization, transport, rescue and mediation, sufficiency, and quantitative-threshold testing. We apply a when–where–how framework to four cases. Perturbations of T6P, SnRK1, and TOR support T6P-dependent kinase regulation as a mediator of lateral-root development and are consistent with a context-dependent candidate gate, but its window and threshold remain unresolved. Circadian regulation of *AHA3* and *SUC2* by CCA1 provides the most complete chain, combining cell-specific perturbation, transport, and rescue. The *OsARF18–OsARF2–OsSUT1* pathway supports sucrose transport as a mediator of rice fertility, although receiving-cell necessity and quantitative restoration remain unresolved. H^+^-ATPase perturbations identify apoplastic pH as a proximal mediator of *Arabidopsis* hypocotyl and cotton-fiber elongation, with context-dependent optima rather than a shared threshold. Nutrient and redox regulation, specialized metabolism, stress, and senescence provide boundary comparisons. Causal confidence depends on claim-matched evidence for responding cells and developmental windows and, where relevant, transport routes, mechanisms, and whole-plant trade-offs. The framework can guide AI-assisted breeding by prioritizing genotype-, stage-, and tissue-specific interventions for prospective perturbation and rescue.

## 1. Introduction: Evaluating Causal Claims in Plant Developmental Metabolism

Developmental genes specify what a plant cell can become; metabolism helps determine whether and where that program is executed and what outcome it produces. Germination, meristem activation, elongation, differentiation, flowering, fertility, and senescence require carbon skeletons, ATP, reducing power, mineral nutrients, and membrane and wall precursors. These requirements vary with cell identity and stage. A demanding program may remain latent even when its transcriptional machinery is present. Altered nutrient supply or source–sink relations can instead change the timing and extent of an otherwise intact program. The relationship is reciprocal: development organizes the production, movement, storage, and use of metabolites, while metabolic state constrains developmental outcomes [1].

This reciprocity has become experimentally tractable. Glucose–TARGET OF RAPAMYCIN (TOR) signaling can reactivate meristematic growth, trehalose 6-phosphate (T6P) links sucrose status to flowering, and the inositol pyrophosphate InsP8 participates in intracellular phosphate sensing [2,3,4]. Regulated sucrose efflux and phloem unloading show that carbon distribution depends on transport anatomy and transporter activity, not on source abundance alone [5,6]. Carotenoid-derived compounds such as anchorene further show that metabolites assigned to specialized pathways can influence root patterning [7]. These advances have brought metabolism into the causal analysis of development. They have also exposed a recurring problem: metabolic evidence is often described with greater causal certainty than the experiments warrant.

Abundance is not a mechanism. A measured metabolite pool integrates synthesis, consumption, import, export, sequestration, and compartment size; identical pools can conceal different fluxes. A compound that rises during organ maturation may drive the transition, permit it, transmit an upstream input, accumulate as a consequence, or merely report developmental stage. Calling every changing compound a “signal” erases these distinctions. The central task is not to compile metabolites associated with development, but to determine what each compound does and what level of causality the evidence supports [8].

We address this problem by separating two annotations that answer different questions. Biological role describes what a chemical entity does. It may act as a substrate or energy source, transport currency, storage reserve, regulatory messenger, structural or local chemical effector, or defense and ecological product. Causal status describes its position relative to a specified developmental output: driver, permissive condition, mediator, consequence, or marker. Neither annotation is fixed across compartments or developmental windows. Sucrose, for example, can supply carbon, move through the phloem, and engage sugar-sensing pathways, while its accumulation at a particular stage may still be consequential rather than instructive.

**Figure 1:**
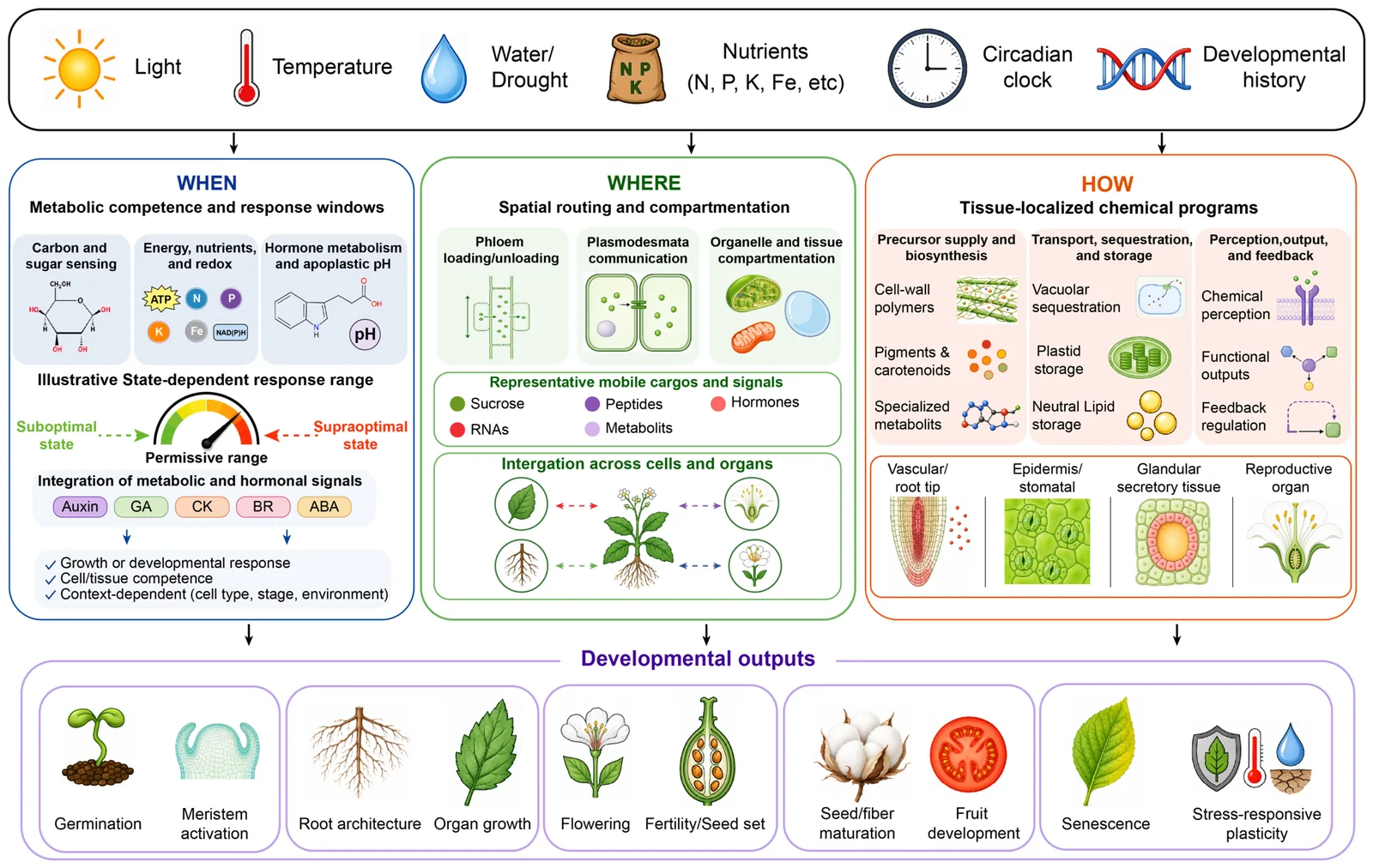
Framework for plant developmental metabolism. The three axes ask when metabolic state permits or changes a developmental program, where material is routed and state is interpreted, and how tissue-localized chemistry executes organ function. Black solid arrows connect representative environmental, temporal, and developmental contexts to the analytical axes and link the axes to developmental outputs. Colored dashed bidirectional arrows in the WHERE panel indicate representative exchange among organs. The axes are complementary analytical perspectives rather than a fixed linear pathway. Abbreviations: GA, gibberellin; CK, cytokinin; BR, brassinosteroid; ABA, abscisic acid. The WHEN gauge is schematic: its permissive range and boundaries are context dependent and do not imply a universal numerical threshold.

We organize the review around three questions (Figure 1; Table 1). First, we ask when a metabolic state changes the competence or response of a defined cell or organ. Second, we ask where the relevant material or state is generated, routed, stored, consumed, and experienced. Third, we ask how local chemistry alters cell behavior, tissue function, or organ performance. These questions overlap and are not consecutive steps in a universal pathway. Their value is operational: together they prevent a temporal profile, spatial map, or molecular association from standing in for a complete causal explanation.

The question of when is captured by metabolic competence. We use “state-dependent metabolic gate” for a context-dependent metabolic or energy-coupled physicochemical condition whose effect is restricted to a defined cell type and developmental window. The state must precede the output and remain perturbable and restorable before developmental commitment. Reversibility therefore applies to the metabolic state and pre-commitment response probability, not to a completed morphogenetic event. Evidence that meets only part of this standard supports a candidate gate; evidence for necessary supply alone supports a metabolic constraint. A threshold is narrower still: it requires a reproducible change point in a defined input–output relationship. *Arabidopsis* hypocotyl and cotton-fiber experiments instead reveal context-dependent response optima with operational low-pH boundaries; they do not establish a universal pH gate or numerical threshold [9,10].

Where directs attention to spatial organization. Phloem loading and unloading, plasmodesmal connectivity, transporter turnover, organellar compartmentation, and storage anatomy determine whether a metabolite acts within one cell, across a tissue boundary, or systemically. Transport of material must be distinguished from transmission of information: movement of sucrose or phosphate does not itself show that a distant tissue interprets that movement as a developmental signal. Local perturbation, grafting, reciprocal rescue, and cell-resolved measurements can separate these possibilities.

How concerns the chemical execution of development. Cell-wall polymers, membrane lipids, pigments, apocarotenoids, defense metabolites, and secretory products can contribute directly to expansion, mechanics, protection, or organ function. We use “tissue-localized chemical program” for this broad category and reserve “circuit” for cases in which production, transport, storage, target engagement, and feedback have been connected experimentally. This caution is especially important in specialized metabolism, where pathway co-expression or stage-specific accumulation often precedes evidence for developmental function [11].

These organizing questions build on established work showing that T6P and SnRK1–TOR couple metabolic state with plant growth and development [12,13,14]. We use them here to state and test causal claims for defined developmental processes.

The framework specifies the developmental window, metabolic variable, cellular or subcellular location, intervening process, and developmental output. It also separates biological role, causal status, evidential support, and whole-plant trade-offs. This plant-centered formulation accommodates changing source–sink identities, phloem and plasmodesmal routing, cell-wall and apoplastic mechanics, organellar compartmentation, and specialized secretory or storage anatomy.

### Scope and Organization of the Review

We examine four core cases in depth rather than provide a systematic inventory. The four cases are: (1) T6P–SnRK1/TOR control of lateral-root development; (2) circadian *CCA1–AHA3/SUC2* control of phloem pH, sucrose loading and transport, and root growth; (3) *OsARF18–OsARF2–OsSUT1* control of reproductive sucrose delivery; and (4) H^+^-ATPase-dependent control of apoplastic pH in *Arabidopsis* hypocotyls and cotton fibers. These four cases are compared in Section 7.1. Other studies are used briefly to illustrate confounders, missing evidence, or limits to transfer across tissues, species, and environments. Descriptive omics studies identify candidates rather than establish causality. Hormone, light, chromatin, and protein-turnover studies are included only when they help define or interpret a metabolic variable relevant to the framework. Examples were drawn from peer-reviewed literature available through July 2026. The aim is to derive discriminating judgments and decisive experiments from a small set of mechanisms, not to catalog developmental metabolites.

## 2. A Causal Framework for Developmental Metabolism

Plant metabolism is inseparable from development, but “developmental information” should be used as an operational description rather than an intrinsic property of a molecule. We consider a metabolic contribution regulatory when altering it changes the probability, timing, rate, or direction of a specified developmental outcome. Such regulation operates through a sensor or a reproducible physicochemical response. This differs from metabolic support, which makes growth or differentiation feasible by supplying energy and material. It also differs from chemical execution, in which local products build or modify walls, membranes, barriers, pigments, or defensive structures. All three can be causally important; only the first requires the stronger claim that metabolic state is being interpreted to regulate development. A causal claim should specify whether the variable is a chemical entity, a local state such as pH or redox balance, or a process such as flux or transport. Pool size alone cannot establish a process.

### 2.1. Biological Role and Causal Status Are Independent

Chemical entities participate in development through six distinguishable but nonexclusive biological roles, and the assigned role may change with compartment, developmental window, and the output being explained. First, they serve as substrates and energy sources. Sucrose imported into developing seeds [15], fatty-acyl derivatives used to construct pollen walls [16,17], and nucleotide sugars used for wall assembly [17,18] are indispensable materials. Their depletion can limit development without showing that the molecules are sensed as developmental signals. Second, entities can act as transport currencies. Sucrose, amino acids, and mineral nutrients move between source and sink tissues. Developmental consequences can arise from the amount and direction of transfer rather than receptor-mediated signaling [5,6,15,19]. Third, starch, storage lipids, and proteins buffer supply across daily or life-cycle transitions. The temporal control of *Arabidopsis* starch use illustrates why reserve function cannot be inferred from a static pool alone [20,21].

Fourth, some metabolites are regulatory messengers. This designation is strongest when a compound has a defined molecular target, lies within a physiologically effective range, and changes development when its production or perception is perturbed. T6P, for example, is present at far lower abundance than sucrose but conveys information about sucrose availability partly through inhibition of SNF1-related protein kinase 1 (SnRK1). Molecular-dynamics simulations based on AlphaFold models, supported by mutagenesis and in vitro phosphorylation assays, identified and tested a T6P-binding pocket on the SnRK1 catalytic subunit KIN10. The resulting model proposes that T6P restricts activation-loop reorientation and thereby reduces GRIK1-dependent phosphorylation of KIN10 [22]. Endogenous binding and site occupancy in vivo remain to be resolved. Genetic suppression of *tps1* developmental defects by impaired KIN10 function provides complementary evidence that excessive SnRK1 activity is a major consequence of deficient T6P signaling [12,23].

Fifth, chemical entities can act as structural or local effectors by supplying or modifying the material from which tissues are built. In maize, the UDP-glucose 4-epimerase BZU3 controls UDP-glucose availability, guard-cell wall composition, and stomatal morphogenesis while also affecting protein N-glycosylation [18]. Such chemistry can execute tissue form without functioning as a receptor-mediated developmental signal. Sixth, defense and ecological products influence herbivory, pathogens, pollinators, competitors, or autotoxicity. These products can impose developmental costs through precursor demand or specialized storage anatomy. Cotton pigment-gland studies provide a direct example of coupled defensive chemistry and secretory-tissue development [24,25,26]. By-products and developmental markers are not a seventh biological role; they describe causal status. A stage-resolved signature can predict developmental state without regulating it.

Classical hormones occupy a boundary in this classification. They are metabolic products whose biosynthesis, transport, perception, and signaling have evolved into specialized regulatory systems. Hormone pathways are included here when carbon, nutrient, redox, or precursor state alters hormone production, turnover, or sensitivity, or when hormone signaling directly reorganizes metabolic flux [27,28]. A hormone-associated phenotype alone is insufficient to infer metabolic control.

### 2.2. Causal Evidence Is Multidimensional

Evidence for developmental metabolism is multidimensional rather than strictly hierarchical. Co-occurrence of a metabolic variable and a developmental stage establishes association. Spatial colocalization and temporal precedence strengthen a hypothesis but do not prove causality. Targeted loss tests necessity, physiological induction tests sufficiency under stated conditions, transport measurements resolve route, and direct-target or epistasis experiments address mechanism. These dimensions answer different questions; none becomes stronger simply by appearing later in a fixed sequence. Exogenous application also requires caution because uptake, compartmentation, and applied concentration may differ markedly from endogenous physiology.

A causal argument should combine the evidence dimensions relevant to the claim (Table 2). The variable should be measured in the responding cells, together with its dynamics, turnover, or flux when these are relevant to the proposed mechanism [29,30]. Tissue- or stage-restricted loss tests local necessity, whereas complementation from a defined promoter tests spatially restricted rescue. Restoring the candidate state in the relevant context can support mediation; rescue through a downstream effector should be reported separately as bypass evidence because it does not by itself establish endogenous mediation. Dose–response analysis, genetic epistasis, direct-target tests, and separation of catalytic from non-catalytic functions can distinguish a regulator from general metabolic failure. An enzyme transcript is not a metabolite measurement; a pool is not flux; and movement of material is not automatically transmission of developmental information.

### 2.3. Gates, Constraints, and Operational Boundaries

A state-dependent gate changes the competence or response probability of a defined cell population during a restricted developmental interval. Operationally, the metabolic variable must have a stronger or qualitatively different effect in the proposed cell population and developmental window than in other populations or at other times. The state change must precede the output, and targeted perturbation must alter the response. Restoring the state before commitment must also restore response probability. The completed developmental outcome—such as an initiated lateral root—need not be reversible. The term “candidate gate” is appropriate only when evidence indicates that the effect is restricted to, or differs across, a defined developmental window or cell population, although one or more criteria remain untested. Without evidence for temporal or population-specific competence, the factor should instead be described as a candidate mediator or metabolic constraint. A metabolic constraint shows that supply or capacity is necessary but not that competence changes in a restricted window. A response optimum is the value or range of the input that maximizes a continuous response, whereas a quantitative threshold requires a replicated change point with uncertainty. These labels are used throughout the analyses below.

Most current studies identify candidate gates, metabolic constraints, or response optima rather than quantitatively defined thresholds. In *Arabidopsis* hypocotyls, imposed extracellular pH produced a biphasic growth curve. Acidification promoted elongation to an optimum, whereas stronger acidification inhibited growth. Light also changed the initial apoplastic state and the response to auxin [9,10]. The external-pH experiment did not determine the absolute apoplastic pH reached in vivo at each treatment. The evidence therefore defines a context-specific response optimum and an operational inhibitory boundary, not a universal pH cutoff. Cotton fibers likewise exhibit an optimal acidification range. This classification makes a falsifiable distinction between a state that changes competence, a supply constraint, an optimum, and a threshold.

The framework is first applied to T6P–SnRK1/TOR control of lateral-root development, which tests whether a metabolic state alters developmental competence. Other carbon, nutrient, redox, germination, and flowering examples define the limits of this case.

## 3. WHEN: Metabolic Competence and Candidate Gates

### 3.1. Core Case 1: T6P–SnRK1/TOR Control of Lateral-Root Development

Carbon availability becomes developmental information through several partially overlapping sensing systems. HEXOKINASE1 (HXK1) occupies the interface between glucose phosphorylation and glucose-responsive gene regulation. Genetic separation of catalytic and signaling functions showed that HXK1 is not merely the first enzyme of glycolysis. It also participates in glucose sensing that alters growth, photosynthetic gene expression, and hormone responsiveness [31]. HXK1-dependent responses should not be generalized to all sugar effects. Glucose can alter osmotic potential, respiration, and hormone metabolism, whereas sucrose can be interpreted through mechanisms that do not require complete conversion to glucose.

T6P provides a more specific link between sucrose status and developmental competence. Its abundance often tracks sucrose, and T6P–SnRK1 signaling helps determine whether tissues favor biosynthesis or energy conservation [13,32]. Together, the direct KIN10 mechanism and the genetic suppression described above strengthen the molecular and developmental evidence chain for this long-standing model [22,23]. In rice, the sugar-inducible OsNAC23–T6P–SnRK1a feed-forward loop coordinates sugar homeostasis with source-to-sink carbon allocation and increased sink-organ growth, extending this evidence to a crop source–sink and yield context [33]. PIF- and MYB-dependent responses provide boundary comparisons in which carbon status is linked to transcriptional outputs without establishing the T6P-dependent SnRK1–TOR mechanism proposed for lateral-root competence [34,35].

SnRK1 and TOR translate carbon and energy state into broad changes in biosynthesis, translation, catabolism, and growth. Glucose–TOR signaling activates *Arabidopsis* meristems through glycolytic and mitochondrial energy production and E2F-dependent cell-cycle activation, showing that carbon availability can release a dormant meristematic program [2]. SnRK1 favors metabolic reprogramming under carbon or energy limitation [13,36]. These kinases are often presented as a binary anabolic–catabolic switch, but their relationship depends on tissue, time, and input. They should be treated as interacting state variables, not universal mirror images [14,37,38].

Lateral-root formation illustrates why reversibility must be assigned to the pre-commitment metabolic state rather than the completed organ. Genetic, metabolic, and pharmacological evidence indicates that T6P promotes lateral-root development through SnRK1 inhibition, TOR activation, and interaction with auxin-dependent programs [39]. These perturbations establish a functional contribution that exceeds a correlation between sugar content and root number. Founder-cell-targeted manipulation places T6P action within the lateral-root lineage, but the precise pre-commitment window and local T6P, SnRK1, and TOR dynamics remain unresolved [39]. No study has yet shown that restoring the T6P–SnRK1/TOR state before founder-cell commitment restores initiation probability. The case is therefore classified as a candidate gate, not a universal T6P switch. Cortex/epidermis-specific ZmbZIP89–ZmPRX47 control of ROS homeostasis and VAMP721/722-dependent trafficking serve as boundary comparisons. They couple local state or transport to lateral-root growth through distinct mechanisms [40,41].

### 3.2. Boundary Comparisons: Nutrient and Redox Constraints

Nutrient and redox mechanisms provide boundary comparisons rather than additional core cases. Phosphate status is interpreted through InsP8–SPX–PHR networks and spatial allocation, nitrate through NRT1.1/auxin, CPK–NLP, and CEP-mediated systemic demand, and ROS through species- and cell-specific redox mechanisms [4,42,43,44,45,46,47,48,49,50,51,52,53,54]. Together, they show that external availability, intracellular sufficiency, mobile demand signals, and local redox state are separable variables. Because matched developmental windows, local restoration, and quantitative boundaries remain incomplete, these examples support nutrient/redox constraints or candidate local controls—not a shared metabolic gate.

### 3.3. Delimiting the Core Case: Convergent Controls Are Not a Single Gate

Comparisons with germination, root branching, and flowering clarify the limits of the lateral-root claim. Germination integrates ABA/GA balance, reserve mobilization, lipid metabolism, nitric oxide, and reversible signaling [55,56,57,58,59,60,61,62,63,64,65]. Root branching combines T6P, nitrate–auxin, and ROS inputs that act in different cells and stages [39,40,41,47,48,49,50,51,52,53,54]. Flowering integrates T6P with TOR-linked photoperiodic machinery [3,66]. Because no common experiment aligns these inputs in the same responding cells and developmental window, they support convergent controls on competence, not a single distributed gate.

The when analysis therefore yields a restricted judgment: T6P–SnRK1/TOR can alter lateral-root development, but the precise pre-commitment window, responding cell, and quantitative boundary remain incompletely resolved. These gaps are spatial as well as temporal: the relevant metabolic state must be resolved in the cells that initiate the response.

## 4. WHERE: Spatial Routing and Temporal Coordination

### 4.1. Source–Sink Networks Define the Reach of Metabolic Effects

Source and sink identities change with developmental stage, so whole-plant abundance is a poor proxy for the state experienced by a target cell. SWEET-mediated efflux, anatomically selective phloem loading, and regulated unloading show that route and interface must be measured directly [5,6,67,68,69]. These principles frame the two transport cases considered next.

ABA-dependent SWEET phosphorylation and plasmodesmal callose control are compact boundary comparisons. They show that transporter state and symplastic connectivity can redirect allocation. However, they are also confounded by multiple cargoes and systemic effects [70,71,72]. Isotope tracing, metabolite sensors, mobile reporters, and local rescue are therefore needed before a developmental phenotype can be assigned to a specific transported metabolite.

### 4.2. Core Case 2: Circadian Control of Phloem pH and Sucrose Routing

Spatial allocation is scheduled in time. During the day, photosynthesis supplies immediate demand and builds transient starch; at night, starch degradation must sustain respiration and growth until dawn. *Arabidopsis* adjusts starch use to the amount remaining and the anticipated time to dawn. This provides experimental evidence that carbon consumption is temporally programmed rather than determined solely by current abundance [20,21]. The relationship is reciprocal: photosynthetically derived sugars entrain the clock through regulation of clock components, creating a metabolically defined “dawn” [73]. KIN10/SnRK1 and the sugar-responsive transcription factor bZIP63 further adjust circadian phase in response to sucrose [74]. Light- and temperature-responsive clock modules in soybean and *Arabidopsis* provide boundary comparisons for temporal coordination but do not establish sugar-mediated entrainment [75,76,77].

Recent evidence reveals that temporal regulation can be tightly integrated with spatial metabolic control at the cellular level. Cell-type-specific measurements using genetically encoded pH sensors and pH-sensitive dyes uncovered antiphasic oscillations of apoplastic pH between hypocotyl epidermal cells and phloem companion cells [78]. The clock component CCA1 lowered epidermal apoplastic pH and promoted hypocotyl growth, while increasing phloem apoplastic pH. In companion cells, CCA1 directly repressed *AHA3* and *SUC2* transcription. Loading and basipetal phloem transport of esculin, a fluorescent sucrose analogue loaded by SUC2, were reduced, supporting an effect on sucrose loading and transport and thereby linking the pathway to restricted root elongation. Phloem-specific *CCA1* expression was sufficient to inhibit root growth. In the *CCA1*-overexpressing background, *AHA3* overexpression restored phloem pH, esculin transport, and root growth, whereas *SUC2* overexpression alone did not rescue root growth [78].

Among the four core cases, the CCA1 study provides the most complete claim-matched evidence chain. Circadian phase defines the response window, and pH dynamics were resolved in the epidermal and companion-cell apoplasts. CCA1 directly regulates *AHA3* and *SUC2* expression, and SUC2-dependent esculin loading and basipetal phloem transport were measured as in vivo proxies for sucrose loading and transport. Phloem-specific perturbation changes root growth, and *AHA3* overexpression provides rescue in the *CCA1*-overexpressing background [78]. The evidence supports a causal route in which local pH affects distinct processes—epidermal elongation consistent with acid-growth control and energized sucrose loading in the phloem—whose coordination changes whole-plant allocation. Because esculin is a transport proxy rather than a direct measure of endogenous sucrose flux, isotope-based confirmation remains important. The study does not establish apoplastic pH as a universal long-distance signal, and its generality across photoperiods, genotypes, and environments remains to be tested. Mobile clock proteins such as ELF4 coordinate shoot and root timing but are mobile regulators rather than metabolites [79].

### 4.3. Core Case 3: Reproductive Sucrose Routing and Fertility

Reproductive commitment reorganizes source–sink relations before large changes in biomass become visible. FT and Hd3a are mobile proteins that specify floral transition, not metabolic currencies, but their arrival at the apex creates a new pattern of demand. Floral organ growth, pollen production, fertilization, and seed filling then require sustained delivery of carbon and mineral nutrients. For example, NPF transporters in synaptic-like vesicles contribute to iron and copper delivery to seeds [19]. The developmental program and transport system are coupled: reproductive identity creates sink demand, while routing determines whether reproductive development can be completed.

Developing seeds illustrate how transport delimits developmental potential. SWEET-mediated hexose transport is required for normal seed filling in maize and rice, directly linking maternal–filial sugar transfer with endosperm development [15]. Spatial transcriptomics has mapped candidate post-phloem sucrose routes and transporter domains at the maternal–filial interface of maize kernels, but expression domains are not direct measurements of sugar flux [80]. During maize endosperm filling, SnRK1a1 phosphorylates OPAQUE2 at Ser41, reducing its stability and transactivation activity, whereas sucrose relieves this inhibition and supports reserve accumulation [81]. A separate OPAQUE11–ZmSSRP1 module promotes starch biosynthesis in maize endosperm [82]. These examples distinguish the arrival of carbon from its local interpretation and use.

In rice, an OsARF18–OsARF2 cascade connects auxin homeostasis to the sucrose transporter OsSUT1. Excess auxin in the *dao* mutant represses *OsSUT1*, causing radiolabeled sucrose to accumulate in source leaves while delivery to lodicules, anthers, and ovaries declines. *OsSUT1* expression partially rescues floret opening and fertility [83]. This evidence supports sucrose transport as a mediator of fertility within a defined reproductive window rather than merely an association with whole-plant carbon status. The rescue is partial, and receiving-cell-specific necessity has not been demonstrated. The case therefore does not identify the reproductive cell types that require OsSUT1-dependent delivery or the quantitative flux needed for rescue.

Together, the circadian and reproductive cases show why spatial action cannot be inferred from organ-level abundance: the transport route, receiving domain, and time window determine target-cell exposure. Establishing delivery is only part of the mechanism; the next question is how the delivered state changes local cell behavior.

## 5. HOW: Tissue-Localized Chemical Programs Convert Metabolism into Form

A causal account of how metabolism shapes form requires spatial resolution. Sugars, ATP, auxin, and redox cofactors are widespread, yet their effects depend on where they are produced, transported, compartmentalized, and consumed. A tissue-localized program therefore requires a defined metabolic entity, state variable, or process and a spatial boundary or route. It also requires a local biochemical or biophysical mechanism and a measurable consequence for cell behavior or organ function.

### 5.1. Core Case 4: Apoplastic pH as a Proximal Growth Mediator

Cell expansion provides a clear example because metabolic energy is converted into extracellular chemistry. Classical acid-growth and expansin studies established how apoplastic acidification can facilitate wall loosening [84,85]. More recently, cell-surface TRANSMEMBRANE KINASE signaling was shown to promote phosphorylation and activation of plasma-membrane H^+^-ATPases, acidifying the apoplast and supporting auxin-induced hypocotyl elongation [86]. This rapid pathway operates alongside nuclear TIR1/AFB–SAUR signaling, which sustains H^+^-ATPase activation. The output is determined not by auxin concentration alone, but by the capacity of a responding cell to generate and maintain an appropriate extracellular pH.

Direct pH measurements and pH-manipulation experiments refine the conventional “more acidification, more growth” interpretation. In *Arabidopsis* hypocotyls, increasing auxin acidified the epidermal apoplast, whereas elongation followed a biphasic response. Moderate acidification promoted expansion, but stronger acidification inhibited it. Externally imposed low pH identified a transition from growth promotion to inhibition, although the actual apoplastic pH corresponding to the external-medium boundary was not determined [9]. The evidence therefore supports a context-dependent acid-growth optimum and an operational low-pH boundary, not a universal pH switch. In roots, cell-surface and intracellular auxin pathways generate distinct H^+^ fluxes, and rapid alkalinization contributes to growth inhibition rather than elongation [87]. Apoplastic pH is consequently a locally regulated state variable whose effect depends on tissue and developmental context.

Cotton fiber provides a crop-level test of the same local physicochemical mechanism. Each fiber is a single ovular epidermal cell that must maintain turgor, import carbon, expand its plasma membrane, and remodel a primary wall while attaining exceptional length. Earlier work established roles for reversible plasmodesmal gating, sucrose and K^+^ transport, and sucrose synthase during fiber development [88,89]. Chemical-modulator and CRISPR–Cas9 experiments then showed that plasma-membrane H^+^-ATPase activity is required for fiber apoplastic acidification and elongation. Loss of *GhHA4A* or *GhTMK3A* elevated apoplastic pH and shortened fibers, whereas insufficient and excessive acidification both inhibited growth [10]. Spatiotemporally calibrated H^+^-ATPase activation increased fiber length without detectable penalties in the fiber and seed traits measured under the tested conditions. The cotton and *Arabidopsis* data therefore define tissue-specific response optima and operational inhibitory boundaries, with apoplastic pH acting as a proximal mediator of elongation. These studies do not define a reversible developmental gate or a shared numerical threshold.

Cotton-fiber multi-omics provides complementary evidence for this pH mechanism. Spatial transcriptomics, single-cell transcriptomics, and mass-spectrometry imaging identified localized lipid-, polyamine-, and hormone-associated signatures, and functional analysis supported a role for GhBEE3 in fiber initiation [90]. Studies of GhMYB30D04–GhHD1, brassinosteroid–gibberellin interaction, GhACS6, GhGASA10-1, and ABA-responsive proteins further connect membrane production, hormone metabolism, and carbon-use programs with fiber development [91,92,93,94,95]. These studies identify candidate processes and genetically required factors but, apart from the explicitly tested exceptions, do not establish cell-resolved flux, a local mechanism, or rescue in the relevant tissue and developmental window.

### 5.2. Boundary Comparison: Plastid-Derived Regulators and Specialized Metabolic Anatomy

Plastid-derived regulators provide a compact test of mechanistic specificity. Anchorene, β-cyclocitral, and zaxinone affect root or hypocotyl development through distinct relationships with auxin, ABA, or strigolactones [7,96,97,98]. Together, these examples show that a shared biosynthetic origin does not imply a shared export route, receptor, or causal mechanism.

Specialized metabolic anatomy tests whether localization is sufficient for causal inference. Cotton gland studies connect GoPGF-centered regulation with gland formation and gossypol production, while cell-specific perturbations begin to separate anatomy from chemical output [24,25,26,99,100,101,102,103]. These data support coordinated but partly separable control; they do not make gland-associated abundance alone a developmental mechanism.

*Catharanthus* and *Artemisia* single-cell studies resolve multicellular pathway partitioning and identify candidate transport or biosynthetic nodes, whereas WRI5a-dependent lipid transfer provides a related intercellular exchange example [104,105,106,107,108]. These atlases localize candidate pathways, but do not establish flux or causal control.

### 5.3. Boundary Comparison: Fertility Requires Local Chemical Execution

Reproductive-wall chemistry provides a final boundary comparison between delivery and local execution. Pollen and seed development require both imported resources and local wall-building reactions [15,16,19,80,81,82,83]. Rice DPW has fatty acyl–ACP reductase and UDP-glucose 4-epimerase activities, but existing mutations disrupt both activities; separation-of-function alleles are needed to assign their individual contributions [17]. Additional pollen-wall studies reinforce that local assembly affects fertility and stress resistance [109,110,111,112]. Together, these studies show why transport, metabolic competence, and local chemical execution must be tested separately.

The pH case identifies a proximal mediator of elongation, whereas specialized metabolic anatomy and reproductive-wall chemistry mainly reveal the need for separation-of-function tests. Whether these mechanisms retain the same role under stress depends on how environmental conditions shift response windows, transport routes, and local chemistry.

## 6. Stress and Senescence as Boundary Tests of Framework Generality

Stress and senescence test whether the response window, transport route, local mechanism, or whole-plant trade-off identified under favorable conditions shifts when water, oxygen, nutrients, redox state, or source–sink demand changes.

Three compact examples illustrate how individual framework coordinates can shift. ABA/SnRK2-dependent SWEET11/12 phosphorylation redirects sucrose toward roots under osmotic stress, altering the route [70]. Energy-dependent TOR control of RAP2.12 modifies hypoxia-responsive transcription, but does not establish a quantitative developmental gate [113,114,115]. Light changes the starting apoplastic state and thus shifts the auxin–pH growth optimum [9]. These examples test route, competence, and local execution separately.

Senescence tests allocation cost. Jasmonate/redox and phosphate-dependent pathways regulate senescence, and cell atlases map candidate remobilization states [116,117,118], but expression maps do not measure successful nutrient transfer into reproductive sinks [119]. The relevant outcomes are therefore carbon and nutrient delivery, yield, product quality, and trade-offs among competing sinks—not senescence markers alone.

Accordingly, constitutive stay-green, accelerated-senescence, or stress-allocation strategies cannot be assumed to generalize. Interventions should target timed and localized mechanisms rather than permanently activate a broad stress program, and their evaluation should include biomass, reproduction, product quality, and remobilization efficiency across fluctuating environments.

These examples show that the proposed mechanisms must also be tested under stress and changing source–sink conditions, with benefits assessed against competing sinks. These comparisons define the evidence needed to move from mechanistic interpretation to targeted intervention.

## 7. From Association to Causal Inference and Mechanistically Constrained Engineering

### 7.1. Point-by-Point Application of the Framework to Four Core Cases

Table 3 compares the four core cases using the Table 2 codes: A, association; N, necessity; L, localization; T, transport; R, rescue and mediation; S, sufficiency; and Q, quantitative threshold. The symbols +, ±, −, and N/A mean established, partial, not established, and not applicable, respectively. Profiles identify claim-specific support and gaps rather than rank study quality.

**Figure 2:**
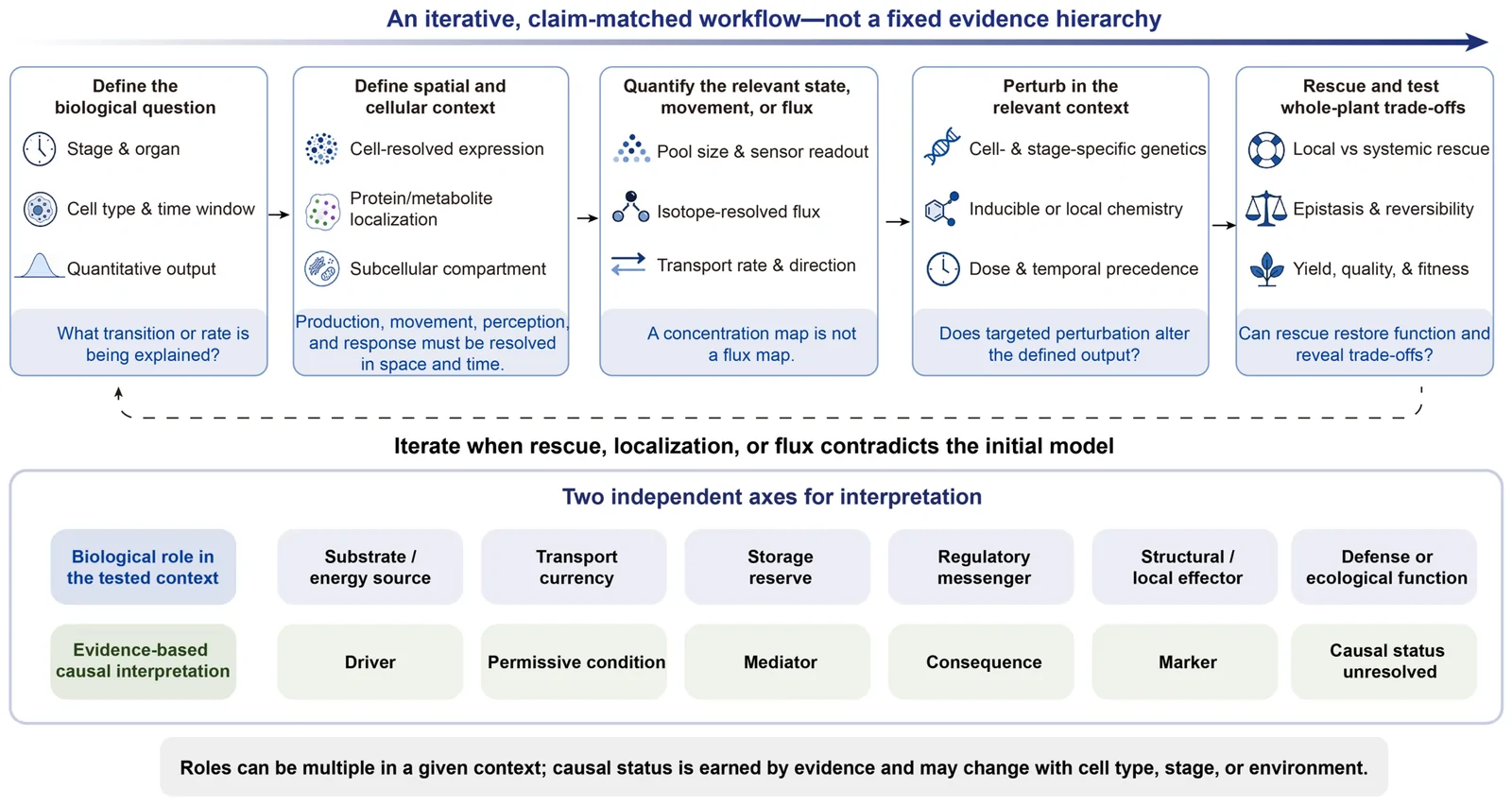
A claim-matched, iterative workflow for causal inference in developmental metabolism. The **upper** panel shows a practical study-design workflow rather than a mandatory evidence hierarchy or fixed sequence. The relevant dimensions depend on the causal claim; state, movement, and flux should be measured only as appropriate to that claim. The dashed return arrow indicates that rescue, localization, or flux results can require revision of the initial model. The **lower** panel shows two independent annotations: biological role and evidence-supported causal status. Vertical alignment does not imply correspondence. “Causal status unresolved” denotes an evidence state rather than a sixth causal category. A chemical entity may have more than one biological role, whereas its causal status is context dependent.

Among the four cases, CCA1 has the most complete profile, although endogenous sucrose flux is unmeasured [78]. T6P evidence supports a candidate gate with unresolved timing, local state, rescue, and threshold [22,23,39]. OsSUT1-dependent transport is an incompletely localized mediator [83], whereas apoplastic pH is a proximal mediator with a context-dependent optimum [9,10,86]. These gaps motivate Section 8.2; Figure 2 summarizes the workflow.

### 7.2. Measure Dynamic State and Flux in the Relevant Cell

Biosensors are most informative when they measure the proposed variable at the scale of the response. Ratiometric imaging with 8-hydroxypyrene-1,3,6-trisulfonic acid (HPTS) in *Arabidopsis* hypocotyl epidermal cells [9] and HPTS-based measurements of cotton-fiber apoplastic fluid [10] relate extracellular pH to growth rather than inferring acidification from bulk-medium measurements. Fluorescent biosensors, including reporters for sugars, ATP, and redox state, can reveal whether neighboring cells experience different inputs [29,30]. However, they require in situ calibration and controls showing that reporter expression does not buffer the measured compound. A single endpoint image is insufficient for a candidate gate claim; the sensor should capture the state before and during the developmental response.

Spatial metabolomics and single-cell transcriptomics solve a different problem. Mass-spectrometry imaging reveals where a compound accumulates, while single-cell or single-nucleus sequencing identifies cells equipped to synthesize, transport, or respond. The 2025 *Arabidopsis* life-cycle atlas includes more than 400,000 nuclei together with paired spatial data across ten stages, providing a broad scaffold for developmental comparisons [120]. A spatially resolved multi-omic atlas of soybean development and the *Arabidopsis* senescence atlas extend this approach to crop organogenesis and nutrient reallocation [118,121]. Single-cell and spatial profiling of soil-grown rice roots further revealed cell-type-specific wall and defense responses and phloem-derived ABA control under compaction, underscoring how the physical growth environment alters cellular state [122].

Spatial resolution does not guarantee mechanistic resolution. Spots can contain several cells, ionization efficiency varies by tissue, and isomers may be unresolved. Transcript abundance cannot establish enzyme activity or transport direction. Noninvasive chemical exchange saturation transfer (CEST) magnetic resonance imaging provides spatial contrast from exchangeable protons associated with sugar- and amino-acid-rich pools in living sink organs. This approach permits repeated phenotyping without destructive sampling [123]. The contrast may contain contributions from more than one compound and remains pool-sensitive; it does not identify a unique metabolite, turnover rate, or flux without calibration and orthogonal chemical measurements.

Stable-isotope tracing adds information about route, turnover, and temporal order. A ^13^CO_2_ pulse delivered to a source leaf, followed by cell- or tissue-resolved isotopologue analysis, can determine when newly fixed carbon reaches a meristem, fiber, gland, or grain. Local feeding of ^13^C-labeled precursors can distinguish import from local synthesis, while paired ^13^C and ^15^N experiments can track carbon and nitrogen redistribution during senescence or stress. Tracer enrichment is not, by itself, a causal test or a direct flux estimate; quantitative flux inference requires an appropriate kinetic model and measurements over time. Labeling can establish whether redistribution precedes or follows a transition. Causality requires targeted perturbation of the relevant transporter or metabolic step and, where possible, restoration of the affected state [8].

### 7.3. Use Cell-Specific Perturbation and Rescue

Whole-plant mutants are difficult to interpret because central metabolic regulators affect multiple organs and stages. Inducible and cell-type-specific CRISPR interference, activation, or complementation can test whether a candidate acts where the phenotype originates. This logic is illustrated by cell-specific pH and transport perturbations, reproductive-transporter rescue, and spatially partitioned specialized pathways [78,83,104]. Enzyme-dead and transport-dead alleles can separate catalytic or transport activity from scaffolding effects. For signaling metabolites, a chemically matched rescue should restore the phenotype at a physiological concentration and within the native window. Rescue by a high, non-physiological dose establishes pharmacological responsiveness, not necessarily endogenous function [9,10,86].

Local and systemic complementation answer different questions. Restoring a transporter in its native receiving cell can identify a relevant site of action and show that expression in that domain rescues the mutant. It does not establish cell-autonomous necessity, which requires selective loss or depletion in the same population [6,78,83]. Complementation from a companion-cell or mesophyll promoter can test whether upstream supply bypasses the defect, provided that promoter specificity, expression level, and cargo movement are measured [70,78]. In a multicellular alkaloid pathway, perturbation in the producing or receiving cell should be paired with measurements on both sides of the boundary [104]. For fiber growth, H^+^-ATPase manipulation is most informative when combined with pH imaging, wall mechanics, and carbon-flux analysis [10].

### 7.4. Translate Mechanisms as Timed Developmental Interventions

A candidate intervention should specify its input and response window and, where relevant, its transport route, local mechanism, and whole-plant trade-offs. Three examples illustrate this point: OsNAC23–T6P–SnRK1a manipulation coordinated source and sink responses across rice backgrounds [33]. A light-activated T6P precursor improved wheat yield when applied during early grain filling [124]. Editing the tomato *SlCDPK27/26–SlSUS3* sugar brake increased fruit sugar concentrations without reducing fruit weight or yield, although the mutants produced fewer and lighter seeds [125]. Together, these studies show that stage, route, and competing sinks—not metabolite abundance alone—determine whether an intervention is useful.

Translational studies should characterize response curves rather than rely on binary endpoints and should test developmental stage, source capacity, sink demand, transporter kinetics, environmental history, and reproductive cost. These comparisons test whether causal predictions hold across genotypes and environments.

### 7.5. Artificial Intelligence as a Causally Constrained Breeding Tool

Artificial intelligence (AI) can improve plant breeding, but useful predictions must go beyond phenotype estimates. Machine-learning methods already support the integration of high-dimensional genotype and phenotype data and genomic selection, although their performance depends on data structure, sample size, and validation design [126,127]. Within this framework, models can integrate genotype, environment, developmental stage, cell-resolved omics, imaging, sensor or tracer data, and field phenotypes. They can then prioritize genotype-by-stage-by-tissue interventions, discover nonlinear response surfaces, and select informative experiments. For the four core cases, a useful model should do more than predict phenotype from bulk-abundance data. It should also predict the response window and, where relevant, the transport route, local mechanism, and trade-offs among competing sinks [33,90,104,118,120,121,122,123,124,125,128].

Feature-importance scores and cross-validation performance do not, by themselves, establish causal or mechanistic validity [126,127]. AI outputs should therefore be treated as ranked hypotheses. Prospective validation requires targeted perturbation, matched controls, dose–response and time–response analysis, and rescue experiments under relevant biological conditions in genotypes and environments not used for training. Active-learning cycles can then update the model where predictions fail. In this role, AI accelerates causal experiment design and precision breeding; it does not substitute for mechanistic evidence.

## 8. Synthesis: Answers to the Three Questions and Experimental Priorities

### 8.1. What the Four Core Cases Reveal

When. Perturbations of T6P, SnRK1, and TOR support the T6P-dependent kinase state as a mediator of lateral-root development beyond a simple sugar–phenotype correlation [22,23,39]. However, the precise pre-commitment window, local T6P, SnRK1, and TOR dynamics, the effect of pre-commitment restoration, and quantitative response boundaries remain unresolved. The evidence therefore supports a context-dependent candidate gate, not a universal T6P threshold. Glucose–TOR-dependent meristem reactivation provides relevant mechanistic context [2].

Where. The *CCA1–AHA3/SUC2* study links circadian phase and companion-cell pH to SUC2-dependent esculin loading, basipetal phloem transport, and root growth through direct regulation, cell-specific perturbation, and rescue [78]. The rice *OsARF18–OsARF2–OsSUT1* case shows that reduced source-to-reproductive-sink transport contributes to infertility and that *OsSUT1* expression partially rescues floret opening and fertility [83]. Together, these cases show that regulated transport can mediate developmental outcomes even when bulk abundance is uninformative, while highlighting the need to test necessity in specific receiving cells and quantify local exposure.

How. Experiments in *Arabidopsis* hypocotyls and cotton fibers identify H^+^-ATPase-dependent apoplastic pH as a proximal mediator of elongation [9,10,86]. Their biphasic responses support tissue-specific optima and operational inhibitory boundaries, not a shared gate or universal threshold. Across the four cases, confidence increases when evidence relevant to the claim—such as timing, cell type, transport, local mechanism, perturbation, or rescue—converges. The boundary comparisons identify links that remain untested in studies of nutrient and redox regulation, specialized metabolism, stress, and senescence.

### 8.2. Case-Grounded and Cross-Case Translational Predictions

Prediction 1: T6P regulation of SnRK1 and TOR in lateral-root competence. If this pathway acts as a state-dependent gate for lateral-root initiation, matched perturbations across stages and doses should reveal a stage-by-dose interaction in the founder-cell lineage. Verified restoration of the local pathway state before commitment should also restore initiation probability [39]. Similar response curves across stages, or failure to recover initiation despite verified restoration of the pre-commitment state, would weaken the gate interpretation but remain consistent with a broader metabolic constraint.

Prediction 2: Circadian routing. If the root phenotype is mediated by an AHA3-dependent proton motive force, isotope tracing should show that restoring *AHA3* also restores endogenous sucrose delivery to roots on the same timescale as root-growth recovery [78]. If growth and measured carbon flux recover independently, either an additional AHA3-dependent process contributes or the relevant flux has not been fully restored or captured.

Prediction 3: Reproductive transport. Comparing source-phloem-specific and reproductive-tissue-specific *OsSUT1* loss and rescue should identify the relevant site of action. In the effective rescue domain, restored sucrose delivery should covary with recovery of spikelet opening and fertility [83]. Recovery of fertility without measured restoration of local flux would weaken the claim that the rescue re-establishes the endogenous transport mechanism and would raise the possibility of either pathway bypass or incomplete capture of the relevant flux.

Prediction 4: Local pH control. The pH optimum for elongation should vary with tissue identity, developmental stage, light history, and initial apoplastic state, but the relationship between calibrated local pH and wall expansion should be reproducible within each context [9,10]. Only a statistically supported change point, reported with uncertainty and reproduced across contexts, would support a universal threshold; the current evidence instead predicts context-dependent response optima.

Prediction 5: AI-assisted breeding. We hypothesize that models incorporating genotype, environment, developmental stage, tissue identity, transport measurements, local metabolic state, and intervention history will rank interventions that produce reproducible benefits more accurately than otherwise matched models based only on genotype and bulk-abundance data [33,90,104,118,120,121,122,123,124,125,126,127,128]. The comparison should use held-out genotypes and environments, prespecified ranking metrics, and models matched for training-set size, model family or complexity, and candidate-validation budget; differences in data-acquisition cost should be reported explicitly. Candidates selected by both models should undergo the same perturbation, rescue, agronomic, and competing-sink assays.

### 8.3. Concluding Perspective

Metabolic variables can act as drivers, permissive conditions, mediators, consequences, or markers, depending on the claim and context. The four core cases illustrate this distinction. Evidence supports the T6P-dependent kinase state as an incompletely resolved mediator consistent with a candidate competence gate. AHA3-dependent proton motive force and downstream sucrose loading are supported mediators of the root-growth phenotype caused by *CCA1* overexpression. OsSUT1-dependent sucrose transport is supported as an incompletely localized mediator of fertility. Apoplastic pH is a proximal mediator with a context-dependent response optimum.

Progress now depends on measuring state and flux in the relevant cells, perturbing candidate variables within the response window, and restoring the proposed mediator in its native context. AI can help prioritize candidates and design experiments, but feature importance and predictive accuracy cannot replace these causal tests.

Candidate interventions must also be tested at the whole-plant level. A change that benefits one organ but compromises reproduction, defense, or resilience may clarify the mechanism but is unlikely to be useful agronomically. The framework helps specify experiments that could falsify a proposed mechanism and the genotypes, developmental stages, tissues, and environments in which an intervention is likely to remain useful.

## Figures and Tables

**Table 1:** Three complementary coordinates for analyzing developmental metabolism.

Axis	Core Question	Mechanistic Focus	Strong Causal Test	Representative Core Case(s)
When: metabolic competence and response windows	When is a cell or tissue competent to change fate or growth mode?	Sugar and energy sensing; nutrient and redox state; hormone metabolism; apoplastic pH	Establish a restricted response window and temporal precedence; test targeted perturbation and rescue; quantify the input–output curve	Core case 1: T6P–SnRK1/TOR control of lateral-root development
Where: spatial routing	Where is material produced, transported, stored, and interpreted?	Phloem loading and unloading; plasmodesmata; transporter state; organelle and tissue compartmentation	Measure movement or flux; compare local and systemic perturbation or rescue; test transport direction	Core case 2: *CCA1–AHA3/SUC2* phloem loading; core case 3: *OsARF18–OsARF2–OsSUT1* reproductive delivery
How: tissue-localized programs	How does local chemistry support cell identity, mechanics, defense, or organ function?	Precursor supply; synthesis; transport; sequestration; storage; perception and feedback	Disrupt separable program nodes in the native tissue and developmental window; test local rescue and system-level cost	Core case 4: H^+^-ATPase-dependent control of apoplastic pH in hypocotyls and cotton fibers

**Table 2:** Evidence dimensions and claim-matched causal language in developmental metabolism.

Evidence Dimension	Minimum Evidence	Claim Supported	Frequent Confound
Association	Stage-, tissue-, genotype-, or environment-dependent covariation of a measured chemical entity, local state, or process with a developmental output	“is associated with” or “marks”	Cell-composition changes; downstream accumulation
Necessity	Targeted loss or depletion changes the output; tissue- or stage-restricted loss is required for a local-necessity claim.	“is required for”; “is locally required for” only when loss is restricted to the stated domain	Pleiotropic starvation, toxicity, or stress
Spatial localization	Production, accumulation, or sensing is resolved in the relevant cells and time window	“is localized to” or “is enriched in”	Mixed cell populations; non-native expression
Transport mechanism	Movement and direction are measured after targeted transporter perturbation; rate or flux is quantified when the claim concerns transport kinetics or delivery.	“moves from X to Y” or “transport contributes to,” matched to the measurement made	Endpoint accumulation substituted for rate or flux; alternative cargoes or routes
Rescue and mediation	Restoring the candidate state in the relevant context restores the phenotype; rescue through a downstream step is reported separately as bypass evidence.	“supports mediation by” for restoration of the candidate state; “can be bypassed by” for downstream rescue	Systemic compensation; incomplete restoration; non-physiological bypass
Sufficiency	Physiological induction changes the output in an otherwise non-responsive context	“is sufficient under the tested conditions”	Pharmacological overdose; ectopic exposure
Quantitative threshold	Nonlinear dose/time response with a statistically supported change point, reproduced within a defined context and reported with uncertainty	“defines a context-dependent threshold”	Arbitrary cutoffs; missing baseline state or hysteresis test

**Table 3:** Claim-matched comparison of four core cases. Codes: A, association; N, necessity; L, localization; T, transport; R, rescue/mediation; S, sufficiency; Q, quantitative threshold; +, established; ±, partial; −, not established; N/A, not applicable.

Core Case	When	Where	How	Evidence Profile and Causal Judgment	Decisive Missing Test
1. T6P regulation of SnRK1 and TOR in lateral roots [39]	Lateral-root initiation and development are affected, but the precise pre-commitment response window is unresolved.	T6P was manipulated in the lateral-root founder-cell lineage, but local T6P, SnRK1, and TOR dynamics were not measured directly.	T6P inhibits SnRK1 [22,23], supports TOR activity, and interacts with auxin programs during lateral-root development [39].	A+, N±, L±, T N/A, R−, S±, Q−. Evidence supports T6P-dependent regulation of SnRK1 and TOR as a mediator consistent with a candidate gate; the precise response window, local pathway state, rescue, and quantitative threshold remain unresolved.	Cell- and stage-specific dose–response analysis plus restoration of the metabolic state before commitment.
2. CCA1 regulation of *AHA3* and *SUC2* [78]	Circadian phase defines the response window.	Hypocotyl epidermal and phloem companion-cell apoplasts are resolved; basipetal phloem transport links shoot carbon supply with root growth.	CCA1 represses *AHA3* and *SUC2* transcription, increases companion-cell apoplastic pH, and reduces SUC2-dependent esculin loading and basipetal transport.	A+, N+, L+, T±, R+, S+, Q N/A. AHA3-dependent proton motive force and sucrose loading are supported mediators. Cell-resolved pH, phloem-specific perturbation, esculin transport, and *AHA3* rescue converge; endogenous sucrose flux remains unmeasured.	Confirm endogenous sucrose flux by isotope tracing; test robustness across photoperiods, genotypes, and environments; separate pH effects from cargo-specific effects.
3. *OsARF18–OsARF2–OsSUT1* fertility [83]	The period of floret opening defines a vulnerable interval in reproductive development.	Radiotracing shows reduced sucrose delivery from source leaves to reproductive sinks; the receiving cells remain unresolved.	Auxin-dependent repression of *OsSUT1* limits sucrose delivery and fertility.	A+, N+, L±, T±, R±, S−, Q N/A. OsSUT1 is necessary for normal reproductive carbon partitioning and fertility, but its site of action and the extent to which rescue restores sucrose transport remain unresolved.	Source-phloem- versus reproductive-tissue-specific loss and rescue, with time-resolved sucrose delivery and fertility response curves.
4. Apoplastic pH in hypocotyls and cotton fibers [9,10]	Auxin-responsive hypocotyl and fiber-elongation stages define the tested windows.	The relevant state is localized to the epidermal or fiber-cell apoplast.	H^+^-ATPase-dependent apoplastic pH control regulates hypocotyl and cotton-fiber elongation [9,10,86].	A+, N+, L+, T N/A, R±, S±, Q−. Apoplastic pH is a proximal mediator with a context-dependent response optimum, not a universal pH threshold.	Calibrated in vivo pH–growth curves across contexts, followed by restoration of apoplastic pH to the context-specific optimum.

Note: The symbols indicate support for individual components of the stated causal claim and do not constitute an overall ranking of study quality.

## Data Availability

No new data were created or analyzed in this study. Data sharing is not applicable to this article.
